# Broadband terahertz wave generation from an epsilon-near-zero material

**DOI:** 10.1038/s41377-020-00452-y

**Published:** 2021-01-07

**Authors:** Wenhe Jia, Meng Liu, Yongchang Lu, Xi Feng, Qingwei Wang, Xueqian Zhang, Yibo Ni, Futai Hu, Mali Gong, Xinlong Xu, Yuanyuan Huang, Weili Zhang, Yuanmu Yang, Jiaguang Han

**Affiliations:** 1 State Key Laboratory of Precision Measurement Technology and Instruments, Department of Precision Instrument, Tsinghua University, Beijing, 100084 China; 2 Center for THz Waves and College of Precision Instrument and Optoelectronics Engineering, Tianjin University, Tianjin, 300072 China; 3 Shaanxi Joint Lab of Graphene, International Collaborative Center on Photoelectric Technology and Nano Functional Materials, Institute of Photonics and Photon‐Technology, Northwest University, Xi’an, 710069 China; 4 School of Electrical and Computer Engineering, Oklahoma State University, Stillwater, Oklahoma 74078 USA

**Keywords:** Terahertz optics, Metamaterials

## Abstract

Broadband light sources emitting in the terahertz spectral range are highly desired for applications such as noninvasive imaging and spectroscopy. Conventionally, THz pulses are generated by optical rectification in bulk nonlinear crystals with millimetre thickness, with the bandwidth limited by the phase-matching condition. Here we demonstrate broadband THz emission via surface optical rectification from a simple, commercially available 19 nm-thick indium tin oxide (ITO) thin film. We show an enhancement of the generated THz signal when the pump laser is tuned around the epsilon-near-zero (ENZ) region of ITO due to the pump laser field enhancement associated with the ENZ effect. The bandwidth of the THz signal generated from the ITO film can be over 3 THz, unrestricted by the phase-matching condition. This work offers a new possibility for broadband THz generation in a subwavelength thin film made of an ENZ material, with emerging physics not found in existing nonlinear crystals.

## Terahertz radiation: thin film enhancement

A new method for generating terahertz waves exploits the low-permittivity properties of an ultrathin film. Terahertz radiation, falling between the infrared and microwave range, has emerging applications in imaging, communications and materials inspection, and is usually generated through the rectification of optical signals in nonlinear crystals. Yuanmu Yang at Tsinghua University in Beijing and co-workers tried an alternative approach, directing a near-infrared laser onto commercially available films of indium tin oxide just 19 nanometres thick. They observed a large enhancement in the terahertz generation when they tuned the laser to hit the film’s ‘epsilon-near-zero’ state—a point where the permittivity of the thin film becomes vanishingly small. The method is relatively inefficient, but the researchers suggest that it could be enhanced by using other thin films in conjunction with metamaterials.

## Introduction

Terahertz (THz) radiation spanning from 0.1 to 10 THz falls between the microwave and infrared spectral ranges^1^. In recent years, THz technology applications have been rapidly expanding^2,3^ in areas including nondestructive material evaluation^4,5^, imaging^6,7^, sensing^8,9^, and wireless communication^10,11^. THz radiation can be generated through the nonlinear downconversion of optical signals or through the nonlinear upconversion of microwave signals, among which the nonlinear optical rectification method is particularly popular for the generation of broadband THz pulses for spectroscopy-related applications. Despite the recent discoveries of broadband THz generation in air plasma^12,13^ and liquids^14^, THz emission is more routinely generated by pumping solid-state noncentrosymmetric nonlinear crystals, such as ZnTe^15^, GaP^16^, and LiNbO_3_^17^, with a femtosecond laser typically operating in the near-infrared range. However, the intensity and bandwidth of the generated THz signal, as well as the pump wavelength, are often limited by the phase-matching condition in the bulk nonlinear crystal. This problem has recently stimulated growing interest in developing ultrathin THz emitters with thicknesses down to even a few atomic layers. In particular, optical metamaterials composed of split-ring resonators with magnetic dipole resonances have been identified as excellent nonlinear THz sources, with a spectral bandwidth unrestricted by the phase-matching condition, as well as the material absorption in the Reststrahlen region of conventional nonlinear crystals^18,19^. However, the highly sophisticated nanofabrication process and the low laser damage threshold have largely hindered their wide adoption. Nanoscale THz emitters have also been realized with monolayer graphene^20^ and tungsten disulfide^21^, although with limited efficiency. Furthermore, surface THz emission from semiconductors such as InAs, InSb, and GaAs has been investigated^22,23^, although efficient THz emission has only been shown in the reflection configuration.

More recently, materials that exhibit a vanishing real part of their permittivity in certain spectral ranges^24,25^, commonly known as epsilon-near-zero (ENZ) materials, have drawn much attention in the field of nonlinear optics^26–29^ for applications such as second- and third-harmonic generation^30–32^, all-optical switching^33,34^, and tuneable absorption^35^. The enhancement of nonlinear optical responses in materials within a subwavelength thickness, induced by the ENZ effect, can be partially explained by the amplification of the pump laser field due to the continuity of the normal displacement field at the boundary of the ENZ material and the background medium, following the formula *ε*_ENZ_·*E*_ENZ_ = *ε*_0_·*E*_0_, where *ε*_ENZ_ and *ε*_0_ are the permittivities of the ENZ material and background medium, respectively, and *E*_ENZ_ and *E*_0_ are the normal components of the electric fields at the material boundary. When *ε*_ENZ_ approaches zero, the electric field in the ENZ material can be significantly enhanced. The enhanced nonlinearity in the ENZ material can be alternatively explained by the small group velocity, also known as the slow-light effect^36^, in the Ferrell–Berreman mode or the ENZ mode supported in the ENZ thin film.

ENZ effects occur in a wide range of materials, including transparent conducting oxides (TCOs)^27^, doped semiconductors^37^, and polar dielectrics^38^. Indium tin oxide (ITO) is one of the most widely available ENZ materials. It has been used as transparent electrodes in solar cells and consumer electronics. The dispersion of the permittivity of ITO follows the Drude–Lorentz formula $\varepsilon {\mathrm{ = }}\varepsilon _\infty {\mathrm{ + }}\omega _{{\mathrm{p,b}}}^2{\mathrm{/}}\left( {\omega _{{\mathrm{0,b}}}^2 - \omega ^2 - i\gamma _{\mathrm{b}}\omega } \right){\mathrm{ - }}\omega _{\mathrm{p}}^2/\omega \left( {\omega + i\gamma _{\mathrm{f}}} \right)$, where *ε*_∞_ is the high-frequency permittivity, *ω*_p,b_, *ω*_0,b_, and *γ*_b_ are the plasma frequency, resonance frequency, and plasma damping rate of bound electrons, respectively, and *ω*_p_ and *γ*_f_ are the plasma frequency and plasma damping rate of free electrons, respectively^39^. The plasma frequency of ITO further follows the formula $\omega _{\mathrm{p}} = \sqrt {N_{\mathrm{e}}e^2/m_{{\mathrm{eff}}}\varepsilon _0}$, where *N*_e_ is the carrier density, *ε*_0_ is the permittivity in vacuum, and *m*_eff_ is the ensemble-averaged effective electron mass. The carrier density of ITO is typically in the range of 10^20^–10^21^ cm^−3^, leading to an ENZ wavelength *λ*_ENZ_ typically in the near-infrared region.

In this work, we show THz generation from commercially available ITO glass by leveraging the ENZ effect. We observe THz emission from a 19 nm-thick ITO film when pumped around its ENZ wavelength. The bandwidth of the generated THz pulse is ~3 THz, limited only by the bandwidth of the pump laser and the detection crystal. We measure the pump wavelength, power, and polarization dependence to confirm that the THz emission originates from surface optical rectification in the ITO film and is enhanced by the ENZ effect. Moreover, we observe that the most efficient THz generation initially occurs at a pump wavelength slightly blueshifted from the static bulk *λ*_ENZ_ of ITO, which redshifts as the pump fluence increases, which may be associated with the nonlocal effect as well as the unique hot-electron dynamics in the ITO film.

## Results

### Sample characteristics and measurement setup

**Fig. 1:**
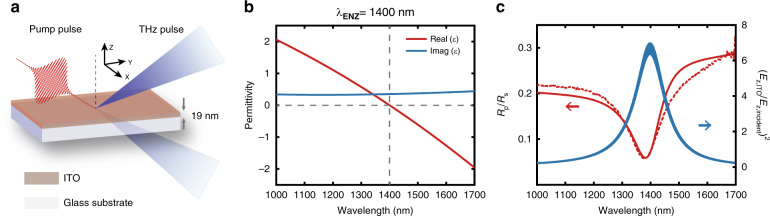
Static optical response of the ITO film. **a** Schematic of the ENZ sample composed of a 19-nm-thick ITO layer and a 1.1 mm-thick glass substrate. **b** Real and imaginary parts of the permittivity of the ITO film as a function of wavelength measured via spectroscopic ellipsometry. The ENZ wavelength is marked with a grey dashed line. **c** *R*_p_/*R*_s_ and (*E*_z,ITO_/*E*_0,incident_)^2^ measured (dashed line) and calculated by the transfer matrix method (solid lines) as a function of the wavelength with an incident angle of 40°, where *R*_p_ and *R*_s_ are the reflectance of *p*-polarized and *s*-polarized light, respectively, and *E*_z,ITO_ and *E*_z,incident_ are the normal components of the electric field in the ITO film and the incident electric field, respectively

A schematic of the commercially available ITO on a glass substrate (PGO GmbH) is depicted in Fig. 1a. The film is found to be amorphous from the X-ray diffraction measurement, with a root mean square roughness of 0.35 nm determined from the atomic force microscopy measurement (see Supplementary Fig. 1 and Supplementary Note 1 for details). The real and imaginary parts of the relative permittivity and the thickness of the ITO film are measured through spectroscopic ellipsometry, as shown in Fig. 1b (see Supplementary Note 2 for details). The real part of its permittivity crosses zero at a wavelength of 1400 nm, with an imaginary part of 0.35. The *p*-polarized linear reflectance spectrum of the sample is measured at an incident angle of 40°, showing a resonance dip near its *λ*_ENZ_ as a result of the excitation of the Ferrell–Berreman mode^25,40^, in close agreement with the calculation, as illustrated in Fig. 1c. The corresponding electric field enhancement in ITO near its *λ*_ENZ_ is also calculated using the transfer matrix method.

**Fig. 2:**
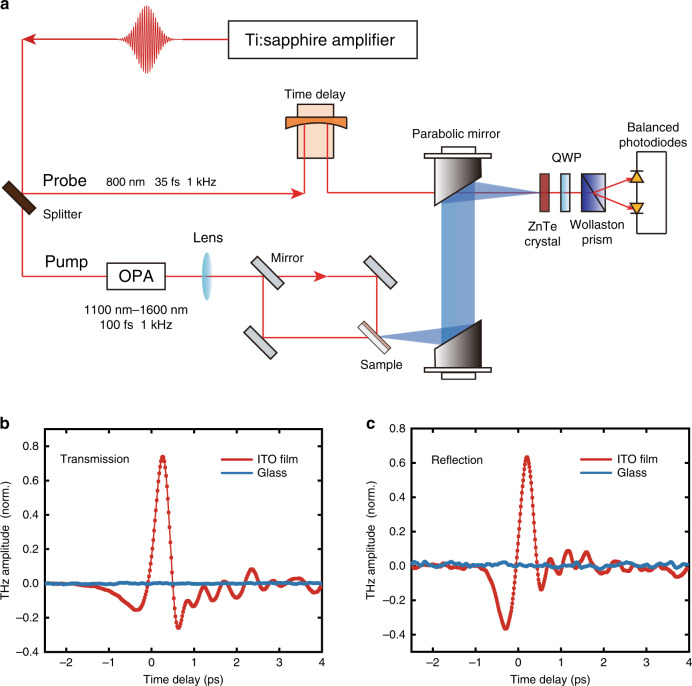
Experimental setup and time-domain THz signals from the ITO film. **a** Schematics of the THz generation and detection setup in both the transmission and reflection configurations. **b**, **c** Time-domain THz signal measured by pumping the ITO film (red) and 0.5 mm-thick bare glass substrate (blue) with pump wavelengths of 1350 and 1400 nm in the transmission and reflection configurations, respectively

The key elements of the THz time-domain emission spectroscopy system in both the transmission and reflection configurations are schematically shown in Fig. 2a. We use the output of an optical parametric amplifier (OPA) to excite the ITO sample with a wavelength tuneable from 1100 to 1600 nm in both the transmission and reflection configurations (see ‘Materials and Methods’ for details). Figure 2b, c illustrate the measured THz time-domain signals. We observe THz emission from the ITO sample in both the transmission and reflection configurations. In contrast, no THz emission is observed from a bare glass substrate under identical optical excitation conditions. We attribute the origin of the THz generation from the ultrathin ITO film to the surface second-order optical nonlinearity. Similar observations on the surface second-order optical nonlinearity of ITO have been made in previous literature studying the second-harmonic generation of ITO films around their ENZ wavelength^39,41,42^.

### Pump wavelength dependence of the THz generation

**Fig. 3:**
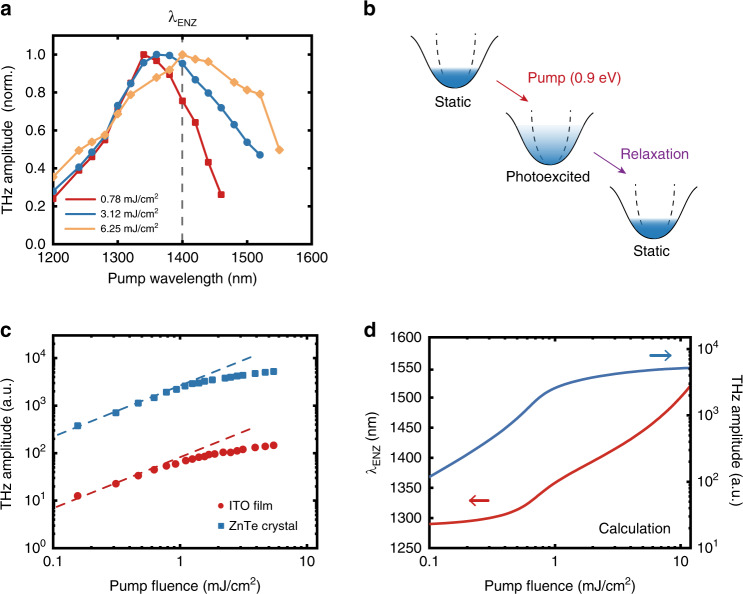
Pump wavelength and pump fluence dependence of THz generation. **a** Measured peak-to-peak THz amplitude as a function of the pump wavelength with pump fluences of 0.78 (red), 3.12 (blue), and 6.25 mJ/cm^2^ (yellow) in the transmission configuration. The dashed curve denotes the static ENZ wavelength of the bulk ITO film measured via spectroscopic ellipsometry. **b** Schematics of ultrafast electron dynamics in the ITO film, which consist of photoexcitation, hot-electron redistribution, and relaxation. The dashed line represents the parabolic conduction band approximation. **c** Measured peak-to-peak THz amplitude as a function of the pump fluence in the transmission configuration with a pump wavelength of 1350 nm for the ITO film (red) and 1 mm-thick ZnTe crystal (blue). The dashed lines are from the linear fitting. **d** Calculated *λ*_ENZ_ of the ITO film (red) and THz amplitude (blue) as a function of the pump fluence in the transmission configuration

To further confirm that the THz emission from ITO is enhanced by the ENZ effect, we measure the pump wavelength dependence of the THz generation. In Fig. 3a, we plot the peak-to-peak amplitude of the THz signal as a function of the pump wavelength, with pump fluences of 0.78, 3.12, and 6.25 mJ/cm^2^ in the transmission configuration. A redshift of the THz generation peak from 1340 to 1400 nm is observed as the pump fluence increases.

At the low pump fluence, the THz generation peak is blueshifted from the static *λ*_ENZ_ of the bulk ITO film measured through spectroscopic ellipsometry. This may be attributed to the nonlocal effect occurring in the ultrathin ITO film, as the electron doping concentration at the surface of the ITO film, where the THz wave is generated, may be higher than that in the bulk ITO film^39,43^. However, as the pump fluence increases, due to the photoinduced heating of conduction band electrons and the consequent time-dependent *λ*_ENZ_ of ITO, the THz generation peak redshifts. As schematically shown in Fig. 3b, upon sub-bandgap photoexcitation, the conduction band electrons in ITO quickly thermalize into a hot Fermi distribution with a maximum electron temperature *T*_e_. Electrons then cool down and relax back to the conduction band minimum through energy exchange with phonons^33,34^. The *m*_eff_ of ITO is a function of the electron distribution and *T*_e_ and is therefore time dependent, owing to the nonparabolicity of the conduction band. According to the Drude–Lorentz formula, an increase in *m*_eff_ leads to a decrease in *ω*_p_ and a redshift in the ITO’s *λ*_ENZ_. The subpicosecond electron dynamics are comparable to the dwell time of the pump pulse inside the ITO cavity, which leads to the pump pulse interacting with a time-variant ITO cavity^44^. As a result, we observe the spectral redshift of the THz generation peak from the static *λ*_ENZ_ of ITO. We develop a quantitative model based on the hot-electron dynamics of ITO to derive a static *λ*_ENZ_ of 1286 nm at the surface of the ITO film, which redshifts to 1340 nm under a pump fluence of 0.78 mJ/cm^2^. Moreover, we calculate *λ*_ENZ_ as a function of the pump fluence (see Supplementary Note 3 for details), which explains the larger redshift when the ITO film is excited with a higher pump fluence and is in good agreement with the literature studying the ultrafast dynamics of ENZ materials^34,44^.

### Pump fluence dependence of the THz generation

To investigate the underlying physical mechanism of the THz generation in the ITO film, we also measure the peak-to-peak THz amplitude as a function of the pump fluence, as shown in Fig. 3c. Under low pump fluence, the THz amplitude scales linearly with the pump fluence, in agreement with the linear scaling law of the second-order nonlinear process of optical rectification. When the pump fluence exceeds 1.8 mJ/cm^2^, the THz amplitude deviates from the linear scaling, which we again attribute to the time-dependent *λ*_ENZ_ of ITO.

For optical rectification, the nonlinear THz polarization is $P_{{\mathrm{THz}}} = \chi _{{\mathrm{eff}}}^{\left( {{\mathrm{THz}}} \right)}E_0^2$, where *E*_0_ is the incident electric field amplitude. We define $\chi _{{\mathrm{eff}}}^{\left( {{\mathrm{THz}}} \right)} = \chi _0^{\left( {{\mathrm{THz}}} \right)}\left( {E_{{\mathrm{ITO}}}/E_0} \right)^2$ as the effective second-order susceptibility of ITO, where $\chi _0^{\left( {{\mathrm{THz}}} \right)}$ is a constant and *E*_ITO_ is the electric field amplitude in the ITO film. It is noteworthy that only the normal component of the electric field in ITO is enhanced under the ENZ condition. Under high pump fluence, the *λ*_ENZ_ of ITO redshifts, leading to lower *E*_ITO_/*E*_0_ and $\chi _{{\mathrm{eff}}}^{\left( {{\mathrm{THz}}} \right)}$. As a result, the THz generation efficiency declines gradually. We calculate the THz amplitude as a function of the pump fluence (see Supplementary Note 4 for details), which qualitatively agrees with the experimental results (Fig. 3d). The THz generation is reversible after repeated measurements with a pump fluence up to 10 mJ/cm^2^, indicating no permanent damage to the ITO film.

In addition, we compare the THz generation efficiencies of the ITO film and a 1 mm-thick ZnTe crystal under identical excitation conditions in the transmission configuration, although the phase-matching condition in the ZnTe crystal is not strictly satisfied at this pump wavelength (Fig. 3c). The thickness of the ITO film is five orders of magnitude less than that of the ZnTe crystal, yet the THz signal is less than two orders of magnitude weaker. The saturation effect in the ZnTe crystal can be attributed to a different origin of multiphoton absorption^45,46^.

### Bandwidth of the THz generation

**Fig. 4:**
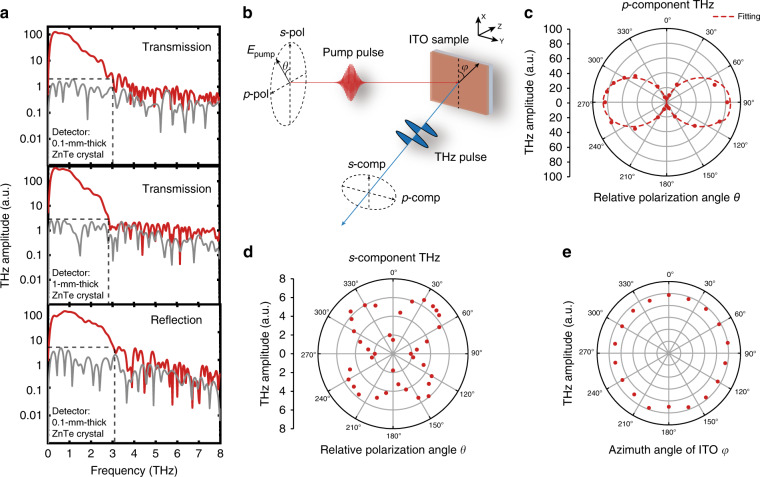
Spectral bandwidth and polarization dependence of the THz generation. **a** THz spectra (red) and noise spectra (grey) generated from the ITO film in both the transmission and reflection configurations, with 0.1 and 1 mm-thick ZnTe crystals serving as the detector. **b** Schematic illustration of the pump light, ITO film, and THz emission. *θ* and *φ* represent the polarization angle and azimuthal angle of the ITO film, respectively. **c**, **d** Polar graphs of the *p*-component and *s*-component THz amplitude as a function of the polarization angle *θ* in the transmission configuration. The *p*-component THz amplitude well fits the sin^2^(*θ*) function, indicated by the dashed lines. **e** Azimuthal angle *φ* dependence of the THz generation in the transmission configuration

The generated THz spectra are obtained by the fast Fourier transform for various pump configurations and detection crystals, as shown in Fig. 4a. We observe a bandwidth of ~3 THz in all cases. In principle, the thickness of the ITO film is deep subwavelength with respect to the pump light such that it does not suffer from the THz generation bandwidth limitation imposed by the phase-matching condition. However, the pulse duration of the pump laser is approximately 100 fs, as measured by autocorrelation, corresponding to a bandwidth of only 4.4 THz^47^. Moreover, the detection efficiency of the ZnTe crystal we use quickly declines beyond 3 THz^47^. To verify that this is indeed an issue, we change the ZnTe crystal with a 1 mm thickness to another ZnTe crystal with a 0.1 mm thickness and observe an increase in the measured spectral bandwidth from 2.8 to 3.05 THz.

### Pump polarization and sample orientation dependence of the THz generation

We further measure the peak-to-peak amplitude of THz signals versus the pump pulse polarization angle *θ*, as shown schematically in Fig. 4b. We define *θ* to be 0° and 90° when the pump light is *s*-polarized and *p*-polarized, respectively. As shown in Fig. 4c, the *p*-component of the THz amplitude well fits the sin^2^(*θ*) function, consistent with the fact that the Ferrell–Berreman mode in ITO can only be excited with *p*-polarized light. Moreover, the *s*-component of the THz amplitude is <10% of the *p*-component of the THz amplitude. Therefore, we can determine the normal component of the electric field *E*_z_ to be the main contributor to the THz generation, which is also in agreement with previous literature reporting second-harmonic generation in ITO films^30^. Moreover, we measure the THz amplitude as a function of the azimuthal angle *φ* of the ITO film and find that the THz generation is not sensitive to the azimuthal angle, as expected from its amorphous structure (Fig. 4e). This can be another advantage of ITO film over other crystalline nonlinear crystals where the THz generation efficiency is highly dependent on the sample orientation.

## Discussion

To summarize, we have observed broadband THz emission from commercially available ITO thin films in both the transmission and reflection configurations. We utilize the ENZ effect and the associated pump field enhancement in ITO to boost the THz generation efficiency. Due to the deep subwavelength thickness, the bandwidth of the THz emission from the ultrathin ITO film is over 3 THz, free from the phase-matching condition. Compared with optical metamaterials, the ITO film has a higher damage threshold^19^. Compared with bulk nonlinear crystals, the THz generation from the ITO film is not sensitive to the azimuthal angle. We attribute the origin of the THz emission to the surface optical rectification process. Its unique pump wavelength and fluence dependence may be attributed to the nonlocal effect and the unique hot-electron dynamics occurring in the ITO film. Currently, the THz generation efficiency from the ITO film is still much less than that of pulsed THz sources based on bulk nonlinear crystals, with a relatively low dynamic range on the order of hundreds, which hinders its imminent application. The THz generation efficiency may be further improved, e.g., by using high electron mobility ENZ materials such as CdO, with a lower optical loss and a larger field enhancement^44^, and by coupling the ENZ material to optical metasurfaces^48^. Moreover, although the ITO film is currently pumped by a solid-state OPA, the *λ*_ENZ_ of ITO can be tailored by controlling the doping concentration during film deposition and postdeposition annealing^30,49^. For example, an ITO film with a *λ*_ENZ_ near 1550 nm can be directly pumped by a more compact fibre-based femtosecond laser with a lower system cost.

## Materials and methods

### Optical measurement

In the experiment, we use a Ti:sapphire amplifier system with a central wavelength of 800 nm, a pulse duration of 35 fs, and a repetition rate of 1 kHz as the pump source. The laser beam is divided into two beams by a beam splitter to generate and detect the THz signal. The main portion of the laser beam is used to pump an OPA to produce infrared pulses with a pulse duration of approximately 100 fs and a wavelength tuneable from 1100 to 1600 nm. The infrared pulse can be configured to excite the sample in either the transmission or reflection geometry. The incident angle of the pump beam is fixed at 40° in the transmission configuration and 45° in the reflection configuration. We have calculated the electric field enhancement factor as a function of wavelength and incident angle (see Supplementary Notes 5 and 6 for details). There is no significant difference in the field enhancement factor at 40° and 45° incidence angles. We did not use the larger incident angle due to the finite size of the ITO sample (1 cm^2^) and the space limitations for sample mounting in our current experimental setup. The sample is placed at the focus of a parabolic mirror, and the generated THz wave is focused on a < 110 > cut ZnTe crystal by another parabolic mirror. The other portion of the laser beam from the amplifier system serves as the probe beam to implement electro-optical sampling of the THz signal through the use of the ZnTe crystal, a quarter-wave plate (QWP), a Wollaston prism, and a pair of balanced photodiodes. It is noteworthy that the ZnTe crystal has the highest detection efficiency for the *p*-polarized THz signal. We can obtain the THz waveform by changing the time delay between the generated THz beam and the infrared probe beam. The polarization state of the pump beam is changed by rotating a half-wave plate and a polarizer. In the measurement, the time step is set to be <0.02 ps and the chopping frequency is set at 370 Hz. The integration time of the lock-in amplifier is set at 300 ms, with a sensitivity of 500 μV. The reported THz traces are the averaged results of over three measurements.

### Electro-optical sampling

To detect the waveform of the generated THz signal, a linearly polarized probe pulse with a wavelength centred at 800 nm is sent into an electro-optical ZnTe crystal collinear with the THz pulse. Due to the Pockels effect of the ZnTe crystal, the birefringence induced by the THz electric field changes the polarization state of the probe pulse. When passing through a QWP, the probe pulse evolves into an elliptically polarized pulse. As it passes through a Wollaston prism, the probe pulse is split into two orthogonal components, which are then sent to a pair of balanced photodiodes. The balanced photodiodes measure the intensity difference Δ*I* between the two orthogonal components of the probe pulse, which is proportional to the amplitude of the THz signal *E*_THz_.

## Supplementary information

Supplementary information
