# Supplementary material for: Broadband terahertz wave generation from an epsilon-near-zero material
Source: Light Sci Appl. 2021 Jan 7;10:11. doi: 10.1038/s41377-020-00452-y (PMC7790823; doi:10.1038/s41377-020-00452-y)
Supplement: Supplementary file 1 — Supplementary information [file 41377_2020_452_MOESM1_ESM.docx]

Supplementary Information for

**Broadband terahertz wave generation from an epsilon-near-zero thin film**

**Wenhe Jia^1†^, Meng Liu^2†^, Yongchang Lu^2^, Xi Feng^2^, Qingwei Wang^2^, Xueqian Zhang^2^, Yibo Ni^1^, Futai Hu^1^, Mali Gong^1^, Xinlong Xu^3^, Yuanyuan Huang^3^, Weili Zhang^4^, Yuanmu Yang^1*^, Jiaguang Han^2*^**

*^1^State Key Laboratory of Precision Measurement Technology and Instruments, Department of Precision Instrument, Tsinghua University, Beijing 100084, China*

*^2^* *Center for THz Waves and College of Precision Instrument and Optoelectronics Engineering, Tianjin University, Tianjin 300072, China*

*^3^Shaanxi Joint Lab of Graphene, International Collaborative Center on Photoelectric Technology and Nano Functional Materials, Institute of Photonics & Photon‐Technology, Northwest University, Xi'an 710069, China*

*^4^ School of Electrical and Computer Engineering, Oklahoma State University, Stillwater, Oklahoma 74078, United States*

^†^*These authors contributed equally to this work.*

** Corresponding authors: [ymyang@tsinghua.edu.cn](mailto:ymyang@tsinghua.edu.cn) or [jiaghan@tju.edu.cn](mailto:jiaghan@tju.edu.cn)*

## **Supplementary Figures**


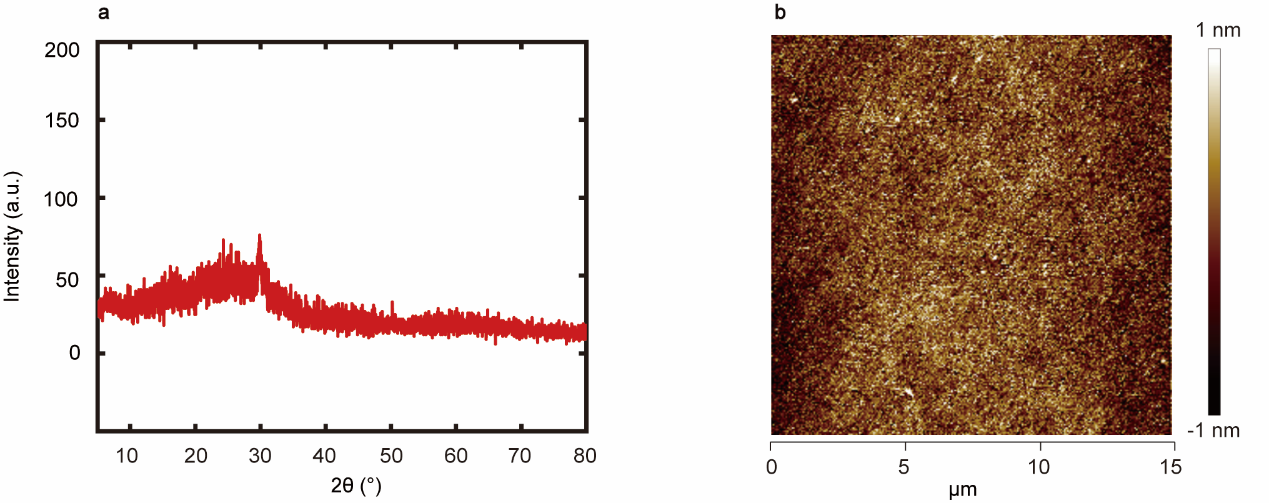


Supplementary Figure 1| (a) X-ray diffraction measurement spectrum of the ITO film. (b) Atomic force microscopy scan of the ITO film. The measured r.m.s roughness is 0.35 nm.


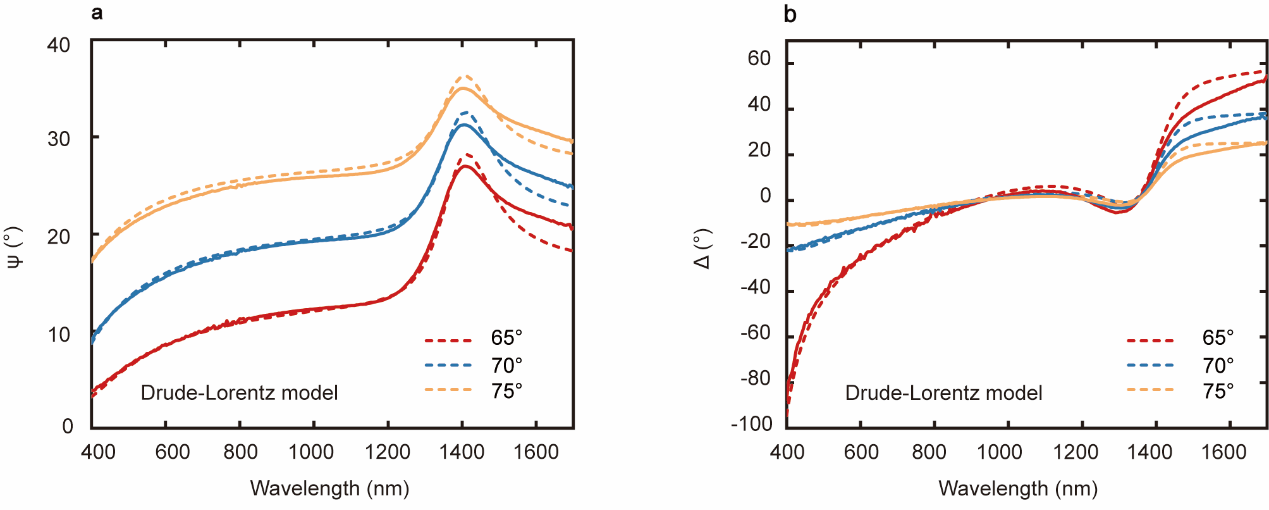


Supplementary Figure 2| (a) and (b) Ellipsometric angles *ψ* and *Δ* of the ITO film measured (solid lines) and fitted with the Drude-Lorentz model (dashed lines) under incident angles of 65°, 70°, and 75°, respectively.


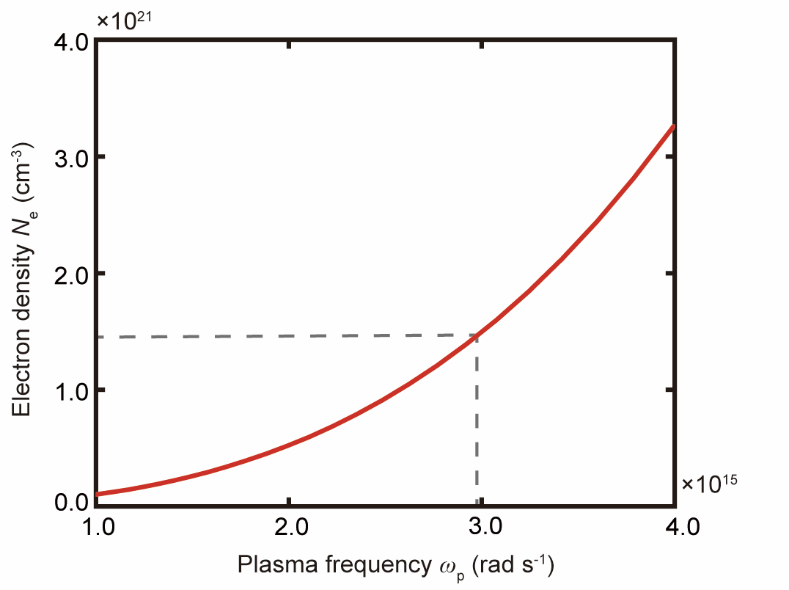


Supplementary Figure 3| The relation between the static (*T*_e_ = 300 K) plasma frequency of free electrons *ω*_p_ and electron density *N*_e_ of the ITO. The dashed lines denote the derived static *ω*_p_ and *N*_e_ of the ITO sample measured in the experiments.
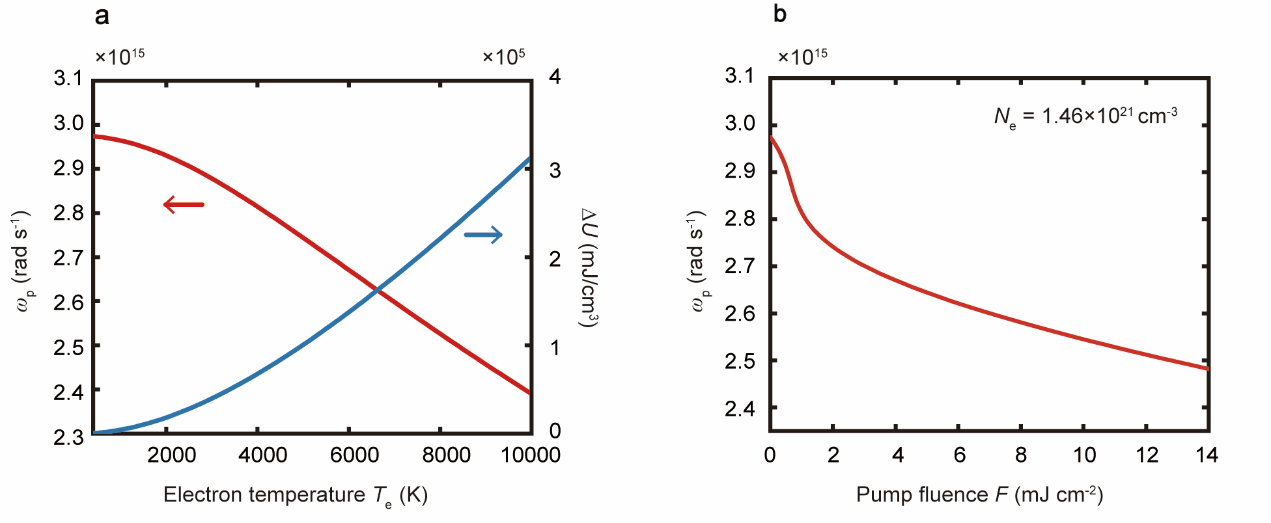


Supplementary Figure 4| (a) The plasma frequency of free electrons *ω*_p_ and the absorbed energy density Δ*U* as a function of the electron temperature *T*_e_. (b) The plasma frequency of free electrons *ω*_p_ as a function of the pump fluence *F*. The calculation is under the assumption of a pump wavelength of 1350 nm, and an incident angle of 40° in the transmission configuration.


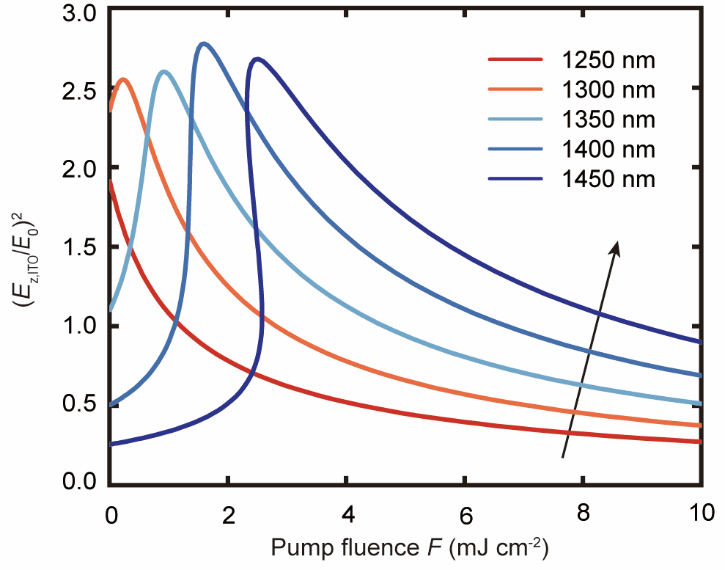


Supplementary Figure 5| The relation between the (*E*_z,ITO_/*E*_0_)^2^ of the ITO film and the pump fluence *F*, under different pump wavelengths, where *E*_z,ITO_ is the normal component of the electric field in the ITO film, and *E*_0_ is the incident electric field.


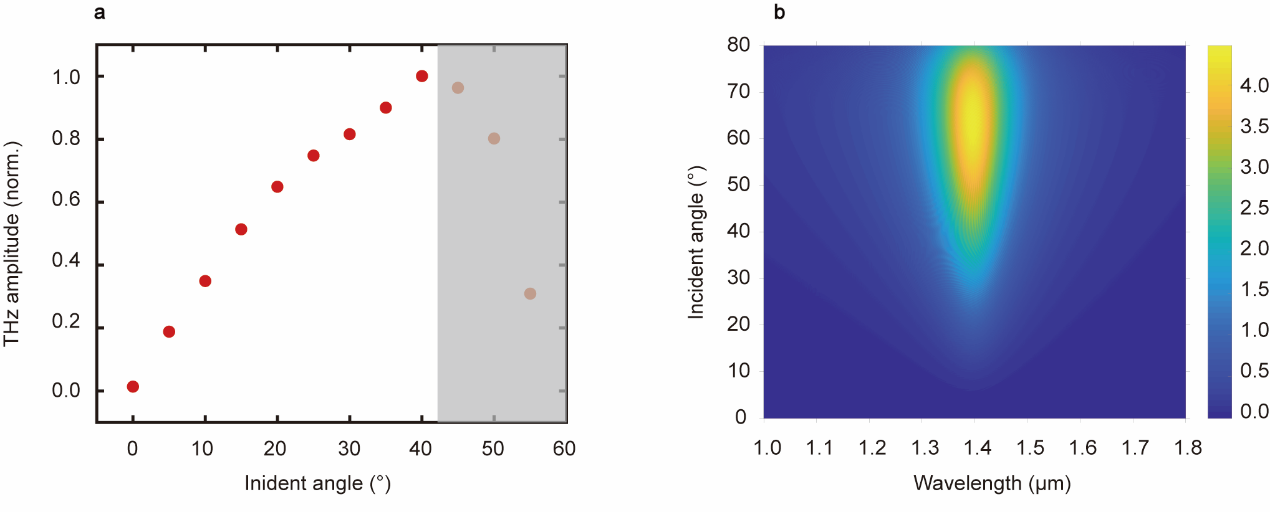


Supplementary Figure 6| (a) The THz amplitude as a function of the incident angle of the pump beam. The grey shadow area corresponds to the case where the incident laser beam is cropped. (b) (*E*_z,ITO_/*E*_0_)^2^ calculated by the transfer matrix method as a function of the wavelength and the incident angle, where *E*_z,ITO_ is the normal component of the electric field in the ITO film, and *E*_0_ is the incident electric field.


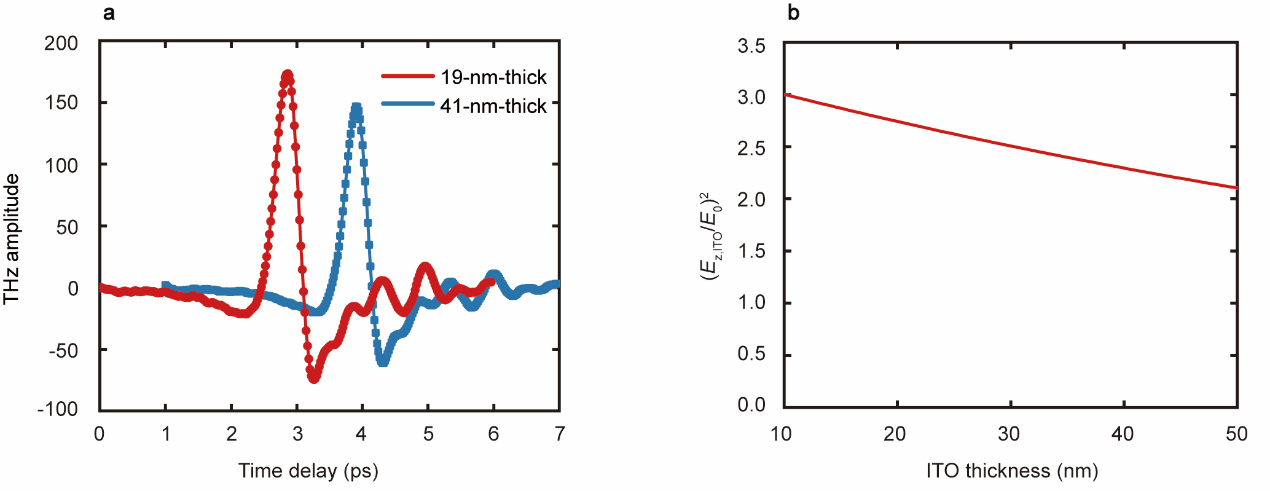


Supplementary Figure 7| (a) Measured peak-to-peak THz signals from 19-nm-thick ITO film (red) and 41-nm-thick ITO film (blue), respectively. (b) The calculated (*E*_z,ITO_/*E*_0_)^2^ of the ITO surface as a function of its thickness under the incident angle of 40°, where *E*_z,ITO_ is the normal component of the electric field in the surface of the ITO film, and *E*_0_ is the incident electric field, assuming the material properties of ITO film with different thickness are identical.

| *ε*_∞_ | *ω*_p_  (rad s^-1^) | *γ*_f_  (rad s^-1^) | *ω*_p,b_  (rad s^-1^) | *ω*_0,b_  (rad s^-1^) | *γ*_b_  (rad s^-1^) | *λ*_ENZ_  (nm) | Thickness  (nm) | MSE |
| --- | --- | --- | --- | --- | --- | --- | --- | --- |
| 2.0956 | 2.7291  ×10^15^ | 5.2496  ×10^13^ | 1.0507  ×10^16^ | 7.4925  ×10^15^ | 3.8176  ×10^15^ | 1400 | 18.803 | 5.35 |

## **Supplementary Table**

Supplementary Table 1| Retrieved fitting parameters of the ITO film with the Drude-Lorentz model.

## **Supplementary notes**

#### **Supplementary note** 1: Characteristics of the ITO film

We take the X-ray diffraction measurement of the ITO film, with the result shown in Supplementary Figure 1a. The lack of a clear diffraction peak in the measurement indicates that the ITO film may be amorphous. We also take a tomography scan of the ITO film using atomic force microscopy, with the result shown in Supplementary Figure 1b. The measured r.m.s roughness is 0.35 nm, which further confirms that the nonlinear optical process in the ITO film is not influenced by undesired surface fluctuations.

#### **Supplementary note** 2: Ellipsometry measurement for permittivity of ITO film

For the ITO film used in the THz generation experiment, its permittivity and thickness are measured via the spectroscopic ellipsometry (J. A. Woollam, Co. V-VASE). We take ellipsometry measurement in the wavelength range of 400-1700 nm under three incident angles (65°, 70°, and 75°), and fit the data with the Drude-Lorentz model:

|  | $\varepsilon\text{=}\varepsilon_{\infty}\text{+}\frac{\omega_{\text{p,b}}^{2}}{\omega_{0\text{,b}}^{2}-\omega^{2}-i\gamma_{\text{b}}\omega}\text{-}\frac{\omega_{\text{p}}^{2}}{\omega\left(\omega+i\gamma_{\text{f}}\right)}$ | (1) |
| --- | --- | --- |

where *ε*_∞_ is the high-frequency permittivity, *ω*_p,b_, *ω*_0,b_ and *γ*_b_ are the plasma frequency, resonance frequency, and plasma damping rate of bound electrons, respectively, and *ω*_p_ and *γ*_f_ are the plasma frequency and plasma damping rate of free electrons, respectively^1^. The measured and fitted ellipsometric angles (*ψ* and *Δ*) of the ITO film are shown in Supplementary Figure 2. The fitted parameters are listed in Supplementary Table 1.

#### **Supplementary note** 3: Non-parabolic conduction band model of ITO film

We attribute the red-shift of the ENZ wavelength *λ*_ENZ_ of ITO to the non-parabolicity of its conduction band. The isotropic and non-parabolic conduction band shape of ITO can be described by Kane’s model as^2^:

|  |  | (2) |
| --- | --- | --- |

where ℏ is the reduced Planck’s constant, *E* is the electron energy with respect to the conduction band minimum (CBM), *k* is the electron momentum, *m*_0_^*^ is the effective mass of electrons at the CBM, and *C* is a coefficient that describes the band non-parabolicity. Notably, the conduction band dispersion is fully determined by *m*_0_^*^ and *C*. When *C* approaches zero, the conduction band recovers to the parabolic shape.

We can obtain the relations among the plasma frequency of free electrons *ω*_p_, the electron density *N*_e_ and the electron energy density *U* of ITO from the linearized collisionless Boltzmann equation^3^:

|  | |  | (3) | |
| --- | --- | --- | --- | --- |
|  | |  | (4) | |
|  |  | | | (5) |

where *T*_e_ is the electron temperature, *μ*_c_ is the electron chemical potential, and *f*_0_ (*μ*_c_*, T*_e_) is the Fermi-Dirac distribution. Here, we use *m*_0_^*^ = 0.263*m*_0_ and *C* = 0.4191 eV^-1^ for ITO, where m_0_ is the free electron mass^4^. We can obtain the static (*T*_e_ = 300 K) *ω*_p_ as a function of *N*_e_ from equations (2) and (4), as shown in Supplementary Figure 3. Due to the conservation of electron density under the intra-band pumping, *N*_e_ is a constant which is independent of *T*_e_. The absorbed electron energy density is defined as:

|  | $\Delta U\left(T_{\text{e}}\right)=U\left(T_{\text{e}}\right)-U(300K)$ | (6) |
| --- | --- | --- |

With photo-excitation (*T*_e_ > 300 K), we can obtain *μ*_c_ (*T*_e_) from equation (2), from which we can further calculate Δ*U* (*T*_e_) from equation (3), and *ω*_p_ (*T*_e_) from equation (4). As a result, we obtain Δ*U* as a function of *ω*_p_. To further verify the experimental results, we need to identify the relation between Δ*U* and the pump fluence *F*. We thus introduce the following formula:

|  | $\Delta U\left(\omega_{\text{p}}\right)=F\cdot A\left(\omega_{\text{p}}\right)$ | (7) |
| --- | --- | --- |

where *A* is the absorption of the ITO sample as a function of *ω*_p_, which can be calculated by the transfer matrix method.

Based on the experimental result that the surface *λ*_ENZ_ red-shifts to 1340 nm under the pump fluence of 0.78 mJ cm^-2^, we first derive the static *ω*_p_ of the ITO surface is 2.9740×10^15^ rad s^-1^, corresponding to *N*_e_ of 1.46×10^21^ cm^-3^. Then, we calculate the *ω*_p_ and Δ*U* as a function of *T*_e_, as shown in Supplementary Figure 4a. Finally, we obtain *ω*_p_ as a function of *F* under the same condition as the experiment in transmission configuration, with a pump wavelength of 1350 nm and an incident angle of 40°, as shown in Supplementary Figure 4b.

Since the dispersion of the permittivity of ITO follows the Drude-Lorentz model, we can calculate *λ*_ENZ_ as a function of the pump fluence *F*, as shown in Fig. 3d in the main text.

#### **Supplementary note** 4: THz amplitude as a function of the pump fluence *F*

According to Figs. 4b-e in the main text, we determine the two independent components of the effective second-order susceptibility tensor of the ITO film: *χ*^(THz)^_zzz_ and *χ*^(THz)^_xzx_ = *χ*^(THz)^_xxz_ = *χ*^(THz)^_yzy_ = *χ*^(THz)^_yyz_. The normal component of the electric field *E*_z_ mainly contributes to the THz generation, which is consistent with the fact that only *E*_z_ is enhanced by the ENZ effect. We then calculate the normalized THz amplitude as a function of *F* (Fig. 3d in the main text) as:

|  | $P_{\text{THz}}=\chi_{0}^{\left(2\right)}E_{\text{z,ITO}}^{2}=\chi_{0}^{\left(\text{THz}\right)}\left(\frac{E_{\text{z,ITO}}}{E_{0}}\right)^{2}E_{0}^{2}\text{=}\chi_{\text{eff}}^{\left(\text{THz}\right)}F$ | (8) |
| --- | --- | --- |

where *P*_THz_ is the second-order nonlinear THz polarization, *E*_0_ is the incident electric field amplitude, and $\text{χ}_{\text{eff}}^{\left( \text{THz} \right)}$ is the effective second-order susceptibility of ITO. Under a low pump fluence, the THz electric field scales linearly with the pump fluence, which is consistent with the linear scaling law of the optical rectification process. However, under a higher pump fluence, the field enhancement is weakened due to the red-shift of *λ*_ENZ_. As a result, the effective nonlinear susceptibility $\text{χ}_{\text{eff}}^{\left( \text{THz} \right)}$ is reduced at the original pump wavelength and the efficiency of THz generation declines gradually.

#### **Supplementary note** 5: Field enhancement factor by transfer matrix method

To calculate the field enhancement factor *E*_z,ITO_/*E*_0_ in the ITO as a function of *F*, we applied the transfer matrix method^5^, in which we denote the medium air, glass, and ITO as layer “0”, “1”, and “2”, respectively.

First, we define the transfer matrix as:

|  | $M_{1}=\frac{1}{2\eta_{1}}\left(\begin{matrix} \eta_{1}+\eta_{0} & \eta_{1}-\eta_{0} \\ \eta_{1}-\eta_{0} & \eta_{1}+\eta_{0} \end{matrix}\right)$ | (9) |
| --- | --- | --- |

where *η*_0_ and *η*_1_ are the admittance of air and the glass layer, respectively. The electric field in each layer can be described (assuming *e*^iωt^ time dependence) as:

|  | $E=Ae^{\text{ikz}}+Be^{\text{-ikz}}$ | (10) |
| --- | --- | --- |

where *A* and *B* are the amplitude of the incident and reflective field, respectively, and $k=\left(2\pi/\lambda\right)n\cos\theta$ is the complex wavenumber. Here *n* is the complex refractive index of the medium, and *θ* is the incident angle. The relation between the incident field amplitude in the air is

|  | $B_{0}=r\cdot A_{0}$ | (11) |
| --- | --- | --- |

where *r*, the amplitude reflectivity at the air/glass boundary, is as a function of *ω*_p_, and can be obtained from the transfer matrix method as well.

For a given electric ﬁeld in the air, we can calculate the electric ﬁeld in the adjacent layer by applying the boundary condition. The tangential electric field in the glass layer at a distance of *d*_1_ from the boundary can be calculated by^6^:

|  | $\left(\begin{matrix} A_{1} \\ B_{1} \end{matrix}\right)=M_{1}\left(\begin{matrix} e^{\text{-ik}_{1}\text{d}_{1}} & 0 \\ 0 & e^{\text{ik}_{1}\text{d}_{1}} \end{matrix}\right)\left(\begin{matrix} A_{0} \\ B_{0} \end{matrix}\right)\cos\theta_{0}$ | (12) |
| --- | --- | --- |

where *k*_1_ is the complex propagation wavenumber in the glass, and *θ*_0_ is the incident angle in the air. According to the equations (8) and (11), we obtain:

|  | $A_{1}=\frac{1}{2\eta_{1}}e^{\text{-ik}_{1}\text{d}_{1}}\cos\theta_{0}\left[\left(\eta_{1}+\eta_{0}\right)A_{0}+\left(\eta_{1}-\eta_{0}\right)B_{0}\right]$ | (13) |
| --- | --- | --- |
|  | $B_{1}=\frac{1}{2\eta_{1}}e^{\text{ik}_{1}\text{d}_{1}}\cos\theta_{0}\left[\left(\eta_{1}-\eta_{0}\right)A_{0}+\left(\eta_{1}+\eta_{0}\right)B_{0}\right]$ | (14) |

The square of the field enhancement factor in the glass layer is then calculated as:

|  | $\left(\frac{E_{z,glass}}{E_{0}}\right)^{2}=\frac{\left\vert\left(A_{1}-B_{1}\right)\tan\theta_{1}\right\vert^{2}}{\left\vert A_{0}\right\vert^{2}}$ | (15) |
| --- | --- | --- |

By repeating the above calculations, we can obtain (*E*_z,ITO_/*E*_0_)^2^ at the surface of the ITO film, where *E*_z,ITO_ is the normal component of the electric field in the ITO film, and *E*_0_ is the incident electric field. According to the equations (10) - (14), we find (*E*_z,ITO_/*E*_z,0_)^2^ is a function of *ω*_p_. Since we have obtained *ω*_p_ (*F*), the relation between (*E*_z,ITO_/*E*_z,0_)^2^ and *F* under specific pump wavelengths at an incident angle of 40° can be obtained, as shown in Supplementary Figure 5.

#### **Supplementary note** 6: Incident angle dependence of the THz generation

To further prove the THz generation from the ITO film is enhanced by the ENZ effect, we measure the THz amplitude as a function of the incident angle under the identical pump power and wavelength, with the result shown in Supplementary Figure 6a. Almost no THz generation can be observed at the incident angle of 0°. As the incident angle increases from 0° to 40°, the THz amplitude from the ITO film increases significantly, which is consistent with the field enhancement calculation, as shown in Supplementary Figure 6b. However, as the incident angle increases further, we observe a drop in the THz amplitude. We believe this is an artifact resulting from the incident laser beam being cropped by the sample mounting fixture at large incident angles.

#### **Supplementary note** 7: THz generation from ITO films with different thicknesses

We have also performed a comparative study of the THz amplitude from ITO films with different thicknesses to prove the THz generation from the ITO film is enhanced by the ENZ effect. We measured the THz amplitude from 19-nm-thick and 41-nm-thick ITO films with a similar doping concentration, under the identical pump fluence, with the result shown in Supplementary Figure 7a. Due to the different thicknesses (nonlocal ENZ wavelength shift) of the two ITO films, we slightly tuned the pump wavelength to maximize the THz generation efficiency of each sample. The THz generation efficiency of the 41-nm-thick ITO film is lower than the thinner one. This result is in qualitative agreement with the calculation showing a lower field enhancement for a thicker sample, as shown in Supplementary Figure 7b.

**References**

1 Rodriguez-Suné, L. *et al*. Study of second and third harmonic generation from an indium tin oxide nanolayer: influence of nonlocal effects and hot electrons. *APL Photonics* **5**, 010801 (2020).

2 Yang, Y. M. *et al*. Femtosecond optical polarization switching using a cadmium oxide-based perfect absorber. *Nature Photonics* **11**, 390-395 (2017).

3 Guo, P. J. *et al*. Ultrafast switching of tunable infrared plasmons in indium tin oxide nanorod arrays with large absolute amplitude. *Nature Photonics* **10**, 267-273 (2016).

4 Liu, X. *et al.* Quantification and impact of nonparabolicity of the conduction band of indium tin oxide on its plasmonic properties. *Applied Physics Letters* **105**, 181117 (2014).

5 Macleod, H. A. *Thin-film optical filters*. (CRC press, 2010).

6 Tocci, M. D. *et al.* Thin‐film nonlinear optical diode. *Applied Physics Letters* **66**, 2324-2326 (1995).
